# What students do when encountering failure in collaborative tasks

**DOI:** 10.1038/s41539-019-0045-1

**Published:** 2019-05-31

**Authors:** Rachel Lam

**Affiliations:** ETH Zürich, LSE Lab, Learning Sciences in Higher Education, RZ J 2, Clausiusstrasse 59, 8092 Zürich, Switzerland

**Keywords:** Social sciences, Education

## Abstract

Experiences of failure can provide valuable opportunities to learn, however, the typical classroom does not tend to function from an orientation of learning from failure. Rather, educators aim to teach accurate information as efficiently as possible, with the main goal for students to be able to produce correct knowledge when called for, in the classroom and beyond. Alternatively, teaching for failure requires instructional designs that function out of a different paradigm altogether. Failures can occur during activities like problem solving, problem posing, idea generation, comparing/contrasting cases, or inventing formalisms or pattern-based rules. We present findings from a study done in fourth-grade classes on environmental sustainability that used a design allowing for failures to occur during collaboration. These center on dialogs that included “micro-failures,” where we could address how students deal with failure during the process of learning. Our design drew from “productive failure,” where students are given opportunities to fail at producing canonical concepts before receiving explicit instruction, and unscripted collaborative learning, where students engage in collaboration without being directed in specific dialogic moves. By focusing on failures during an unscripted collaborative process, our work achieved two goals: (1) We singled out occurrences of failure by analyzing students’ dialogs when they encountered impasses and identified several behaviors that differentially related to learning; (2) We explored how the form of task design influences the collaborative learning process around failure occurrences, showing the potential benefits of more structured tasks.

## Introduction

Experiences of failure can provide valuable opportunities to learn, however, instruction in common school settings tends to be based on student “success.” In such settings, teachers use homework marks, class grades, and exam scores to measure “correctness” of student knowledge; typically the higher the score, the more correct knowledge a student has acquired and therefore, the more successfully s/he has learned. When success is determined by student ability to reproduce correct knowledge as quickly as possible, knowledge accuracy and prevention of mistakes can become unnecessarily prominent.^1^ This can steer the goals of instruction away from a paradigm of learning from failure.

Recent research has shown that students can learn more deeply from failure-based instructional approaches. In contrast, direct instruction such as receiving the “textbook” conceptualizations of topics via lectures or other forms of explicit instruction might merely lead to surface learning.^2^ Generally involving presentation of correct information, direct instruction tends to be teacher-centered rather than learner-centered. When the outcomes of direct instruction are compared to learning activities that allow students to generate their own solutions, ideas, conceptualizations, and representations before being formally taught, the evidence favors the latter for conceptual understanding and ability to transfer knowledge.^3–7^ There is benefit to *delaying* formal instruction,^8^ in part, because it provides opportunities to deal with naive or non-canonical conceptions as forms of “failures” in knowledge correctness.

A few ways that students can encounter failures are by being told that they are incorrect (i.e., a direct instruction strategy), recognizing their incorrect knowledge through task feedback, or discussing their incorrect knowledge with others who are also nascent in the topic at hand. Students have better opportunities to learn from their failures when they recognize them indirectly.^1^ Therefore, rather than direct, our work addresses failure encounters based on indirect feedback via task design and peer discussion using initial naive knowledge. I draw from two major areas of learning research for support: Productive Failure and unscripted Collaborative Learning. Both involve students generating ideas in open-ended task settings, peer-to-peer discussion of ideas, a form of explicit instruction, and indirect feedback on the correctness of generated knowledge.

Productive Failure (PF) is a learning design that follows two main stages of activity.^9^ First, students freely generate solutions, methods, and/or representations in a problem-solving task prior to having the formal knowledge that would typically be considered necessary to successfully complete the task. By design, this leaves sufficient opportunity to encounter failures as the task implicitly gives students information about their knowledge gaps. After this generation period, students receive direct instruction by a teacher that explicitly presents the canonical solutions, methods, and representations. In Kapur and Bielaczyc’s seminal paper on designing instruction for productive failure, they discuss the learning mechanisms that help students to deepen conceptual understanding and increase capacity to transfer knowledge.^4^ The first stage of exploration and generation allows for (a) activation and differentiation of knowledge, (b) attention to critical features of concepts, and (c) explanation and elaboration of those features through student collaboration on the task. The second stage allows for (d) knowledge consolidation and assembly as students attend to the canonical forms of the concepts presented via formal instruction.

Prior work on PF has yet to explore in depth the specific behaviors in which students engage during their experiences of encountering failure. Studies have reported a variety of students’ incorrect solutions to problems and general qualities of student discussions during problem-solving.^9^ However, there has not been PF work that isolates students’ behaviors around and during episodes of failure. This begs the question of what constitutes failure in a learning setting. With failure defined as the inability to produce the canonical representations, solutions, or methods for solving a problem,^4^ the focus lies at the student end-products (e.g., incorrect solutions). Faulty end-products are static forms of evidence that failure has occurred, but are insufficient to inform what students do in the process of dealing with the possibility of having incorrect knowledge. To better understand this requires perceiving failure in a way that becomes visible throughout a dynamic process. Examining dialogs around impasses during a collaborative learning activity is a way to ascertain what students do in the process of encountering failure.

Considering the rich and broad corpus of findings from the Collaborative Learning (CL) literature,^10–16^ I have narrowed the focus here to note (1) two useful design principles for deep learning and the related cognitive mechanisms, and (2) work that addresses how students handle impasses in CL settings. With regard to (1) task design, providing some form of preparation to collaborate through a prior task and balancing the degree of open-endedness in the task have been shown to invoke dialogic behaviors that facilitate learning.^17–23^ An effective preparatory task can immediately provide students with “something to talk about,” priming the cognitive mechanism of prior knowledge activation. This, in turn, can help motivate students to share responses, fostering dialogic behaviors such as explaining, questioning, and debating.^24^ As students continue to engage in discussion, they are likely to self-regulate to reach mutual understanding as each deems necessary in order to complete the task.^25^ Continued activation of prior knowledge can then arise, further motivating the use of effective dialogic moves. In terms of task open-endedness, the extent to which students can generate ideas during preparation is a factor that has differentially influenced both collaborative behaviors and learning outcomes.^26,27^ Some CL work has revealed an interaction between how open-ended the task is and the type of knowledge to be gained.^28^ A more open and complex task tends to be better suited for gaining deep knowledge, as it allows better opportunities for students to collaborate in ways that activate cognitive mechanisms for deep learning.^2^

With regard to (2) student impasses, Tawfik, Rong, and Choi’s theoretical work points to several interconnected failure-based learning designs that illustrate a process of productively handling failure.^1^ They posit that instructional approaches that intentionally incite experiences of failure allow students to challenge their existing (incomplete/inaccurate) mental models or internal scripts. This engages cognitive mechanisms of assimilation as a learner activates prior knowledge, disequilibrium, or cognitive conflict as s/he recognizes the insufficiency of knowledge, an internal state of inquiry while s/he struggles to identify specifics of the knowledge failure, and a restructuring of her/his mental model as new knowledge is identified. The process can be repeated at each recognition of failure. They include peer collaboration as critical to the learning process relative to real-world complex problem solving and preparing students for the future workforce. However, there are additional benefits of collaboration more directly related to the cognitive process in failure-based learning. As mentioned above, collaboration can invoke dialogic behaviors that facilitate learning when contexts are designed with particular features. Engaging in these behaviors increases the chance for knowledge gap/failure recognitions and instances of cognitive conflict. As learners enter an inquiry state, the opportunity to share different perspectives, arguments, questions, and ideas with peers fosters a cycle of continued failure recognitions and attempts to resolve uncertainties. The externalizations of learners’ internal processes via dialogic moves also adds collective knowledge to the collaborative experience while they continue to restructure mental models to resolve failure encounters. In essence, CL contexts provide a space where students can naturally externalize thinking around knowledge failures in dialog, increasing the information available to work with through the generation of ideas, questions, and explanations as they progress towards refining comprehensive and nuanced conceptions. Empirical studies on the process of collaborative learning support these notions.^10,17,18,29^

Tawfik, Rong, and Choi also provide a useful conceptualization of failure for our examination of the experiences that students undergo while learning from failure. They coin the term “micro-failures,” which specifically refer to iterations where a learner recognizes an uncertainty while working through a task and then has the opportunity to respond to improve understanding.^1^ Student dialogs from CL activities provide a way to “see” these iterations, over and above what we glean from student end-products, such as problem solutions.^30,31^ Instructional designs that provide opportunities to experience knowledge failures in collaborative settings may help us to better understand what students do when they encounter, deal with, and attempt to overcome failure. I next briefly describe our design and its theoretical underpinnings from which we drew our data for the current work.

Based on the two stages of Productive Failure and considering the two aforementioned design features of unscripted Collaborative Learning, is a less well-known design called Preparation for Future Collaboration (PFC).^24,32^ In PFC activities, students engage in an exploration task allowing for idea generation and afterward are provided explicit instruction, thus, delaying support towards correct understanding. In general, this follows the typical setup for a productive failure task. What is different is that PFC divides the exploration into two phases: individual exploration followed by peer collaboration. Thus, there are three phases of activity in the PFC design: (1) individual exploration, (2) continued exploration in peer collaboration, and (3) explicit instruction by a teacher. In terms of balancing task open-endedness as a CL design feature, there are still questions around how structured the exploration should be. I come back to this shortly. First, I discuss the cognitive mechanisms that can be invoked across the three phases of PFC.

**Fig. 1:**
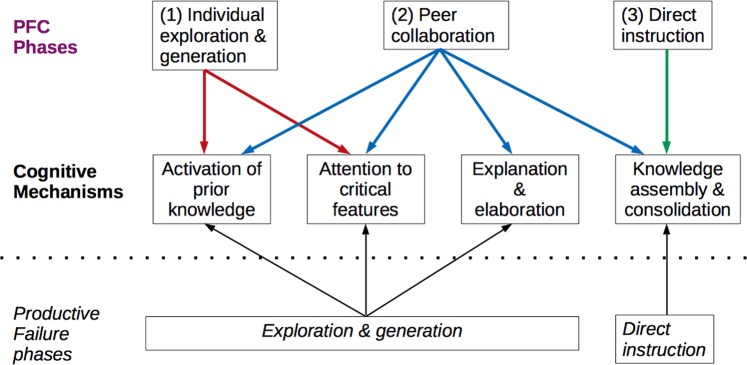
Preparation for future collaboration (PFC) phases and corresponding cognitive mechanisms. The PFC instructional design includes three phases as shown at the top. Arrows point to the cognitive learning mechanisms that are engaged during each phase. Note that multiple mechanisms are invoked in the first and second phases. The two instructional phases of Productive Failure (PF) are shown below the mechanisms. The first phase of PF typically involves group work, thus, invoking the first three cognitive mechanisms. By separating exploration and generation into (1) individual exploration and generation and (2) peer collaboration, as is done in the PFC design, we see a more nuanced representation of how each mechanism aligns to the different phases of learning activity. In both PFC and PF, the mechanism of knowledge assembly and consolidation occurs during direct instruction

The (1) individual preparation phase provides the opportunity for students to activate prior knowledge and attend to critical features of the concepts to be learned by addressing them in different ways. At the (2) collaboration phase, several mechanisms of learning could be engaged. As students discuss their preparatory work, each could elaborate and explain his/her ideas, which allows for further activation of prior knowledge and differentiation of conceptual features. Students might also begin to assemble knowledge during discussion as each aims to refine ideas around the concepts. Finally at the (3) explicit instruction phase, students have the chance to further assemble and consolidate knowledge while the teacher presents the canonical forms of the concepts. The figure below summarizes these cognitive mechanisms for each stage (Fig. 1). Note that these are the same mechanisms in which students engage during a productive failure activity, but occur differently across the PFC phases.^24^

Returning to the degree of task open-endedness, I reference a prior quasi-experimental study we conducted on the overall effectiveness of PFC on learning.^32^ The following details set-up our current work. Our prior study took place in two fourth-grade classrooms of a Singaporean public school. As part of an environmental education curriculum, the students engaged in a complex problem-solving task^33,34^ on the overproduction of waste in Singapore, which is a densely populated urban area. The objective was for students to conceptually understand the “four R’s” of Refuse, Reuse, Reduce, Recycle, and their differences, and identify how to use them to decrease the production of waste on the overall disposal system. The task was set-up according to the stages of PFC. (1) Students first individually worked on question items that implicitly differentiated the four R’s. (2) They then collaborated with a partner and discussed one another’s responses to arrive at a joint decision for each item, and also jointly described how to solve the waste problem. After this, (3) the teacher explicitly presented descriptions and definitions of, and the differences between, the four R’s. This study investigated two versions of open-endedness of the learning task, one being a choice-based selection task and the other highly generative and open-ended. I refer to these versions as Select and Generate. Details on the materials and methods used in our prior study can be found in the Methods section at the end of this paper and the Supplemental Methods file.

In the current work, we examined the entire corpus of student dialogs from the collaboration stage of our prior PFC study in order to discover what interactive behaviors occurred during micro-failures and their relationships to learning. As PFC was developed with failure-based learning in mind, the students from our prior study had ample opportunities to encounter micro-failures while collaborating. The use of two task versions, Select and Generate, allowed us to address how the degree of task open-endedness influenced experiences of failure and the related learning outcomes. I list the main aims as follows:

1. To single out occurrences of micro-failures via dialogs from pairs of students collaborating, and identify the dialogic behaviors within those occurrences.
2. To examine how those behaviors relate to learning.
3. To compare how the degree of task open-endedness (Select vs. Generate) influences the learning process related to micro-failures.

See the Methods section for details on our verbal analyses.

## Results

### Summary of analyses conducted

Analyses were conducted at the dyadic level unless otherwise stated. All available student data were included in analyses (*n* = 40 students, 20 dyads; 16 students were removed due to lack of consent). Learning was measured by posttest scores that were obtained in the prior study. We used the same groupings from the prior study (Select vs. Generate) to assess conditional differences related to micro-failures. In order to further elaborate on the learning processes of our initial findings, we analyzed the behaviors of different groupings of dyads in the two conditions, as well as conducted a contrasting cases analysis of the dialogs of the highest- and lowest-scoring dyads.

### Micro-failures identified

We identified nine categories of behaviors that occurred within failure episodes in the dialogs: questioning and explaining with a partner, explaining after teacher prompts, arguing to consensus, initiating teacher help, making a quick choice without consensus, arguing without consensus, quick arguing with a dominating partner, ignoring contributions and moving forward, and going off task. These were included in the final coding scheme (see Table 1). Two raters then independently coded 20 percent of the total episodes. An interrater reliability check was conducted using Cohen’s kappa and showed sufficient agreement, *κ* = 0.72, *P* < 0.01. Thus, one rater coded the remaining data. We calculated the frequencies of the behaviors. The descriptive statistics of the failure episodes and behaviors are provided in Table 2.

**Table 1:** Coding scheme used to identify behaviors in failure episodes

Behavior code	Description	Example from discourse (*S1, S2* = students/*T* = teacher)
02 Ques/expl–partners	Students are questioning and/or explaining to one another without clear argument/debate (could be for clarification of answers, reasons for answers, or any questions related to the task)	Dyad A S1: This one refuse. S2: Refuse? S1: Ya you can use metal, so you do not need to throw away. S2: Oh yea, do not need to throw away because it is easy to wash.
03 Ques/expl–teacher	Students are explaining or elaborating on their ideas, or providing reasons for answers after being prompted by the teacher.	Dyad B T: [You say] best to reduce food. Why? S1: Because later when you eat, uh… T: Yes, ok. S1: All the food left already, uh… later you, you- S2: Put it in the fridge.
04 Argue–consensus	Students are engaged in an argumentation type of dialog, meaning that they are challenging one another or engaged in debate, and eventually the students come to consensus	Dyad C S1: Why do you think it is recycle? I want reuse because [*explanation*^2^]. S2: Because… inside is the pen right? Er, no. The one is pen and paper? … I choose recycle. S1: You can reuse, you can keep reusing, like using the pen refiller. I think it is reuse. How about you? S2: Ya, I think it is reuse.
05 Initiate teacher help	Students (either partner) initiate teacher help during a failure episode	“Teacher, can you help us?” “Teacher, Teacher! Do not know what to write.”
06 Quick choice–no consensus	Either partner in a dyad quickly names/selects an answer to a question item with very little to no dialog and does not necessarily engage the other to come to consensus	Dyad D^1^ S1: We use tetra packs, we recycle! Of course. S2: Reduce. S1: No, not reduce! Recycle. [*change topic and ignore and move forward - shared number 9 code*^2^]
07 Argue–no consensus	Students are engaged in an argumentation type of dialog, however, they do not ever come to consensus on their disagreement	Dyad E^1^ S1: Refuse. T: Yeah, you agree. S1: Ah no, recycle. S2: Yeah, because they… T: You must ask him if he agrees. S1: Refuse, eh no, recycle. Reuse. S2: You ask me, gotta ask me first! S1: Use less! Reduce! [*prompted by teacher - shared number 3 code*^2^]
08 Quick argue–one dominator	Students engage in a very brief argument, however, one partner generally dominates the interaction and names/selects an answer	Dyad F S1: Reduce or, reduce? S2: I chose recycle. S1: How to recycle bottle? S2: Just put reuse. I think we should put… S1: I have not say my answer.
09 Ignore–move forward	Students (either partner) do not engage around the failure moment but simply move past it	[*see number 6 code above*^2^]
10 Distract	Students (either partner) begin to go off-task and may engage in joking, teasing, singing, talking to other students (non-partners), or otherwise engage in off-task talk	Dyad G S1: Recycle! S2: Refuse. Recycle. S1: Of course la Auntie. You dunno, I am Tommy [the turtle] speaking. S2: Wah, he call me Auntie.

*Notes*: [1] Dyads D and E are examples where more than one code was used. [2] Italics are used to indicate the type of extended dialogue that was removed to save space

This table illustrates the coding scheme used to identify the nine categories of dialogic behaviors that occurred during all micro-failure episodes. It includes the code, description of the code to the right, and real excerpts from the data in the far right column. Dyads are labeled by letters to differentiate the excerpts by code. S1 and S2 refer to the two students in each dyad; they were not the same students across the dyads. Some utterances were removed for the sake of saving space and replaced with their function in the dialog using italics

**Table 2:** Means/SDs of number of coded episodes and behaviors across conditions

	Separated by condition		
Behaviors	Select (*N* = 10)	Generate (*N* = 10)	Total (*N* = 20)
01 Failure episodes	5.40/3.20	3.80/2.49	4.60/2.91
02 Ques/expl–partners	1.70/1.49	1.40/1.71	1.55/1.57
03 Ques/expl–teacher	1.50/3.14	0.90/1.52	1.20/2.42
04 Argue–consensus	1.00/1.05	0.00/0.00	0.50/0.89
05 Initiate teacher help	0.20/0.42	0.30/0.67	0.25/0.55
06 Quick choice–no consensus	0.20/0.42	0.30/0.67	0.25/0.55
07 Argue–no consensus	1.00/1.49	0.60/1.26	0.80/1.36
08 Quick argue–one dominator	0.60/0.84	0.70/0.82	0.65/0.81
09 Ignore–move forward	0.40/0.70	0.06/1.07	0.50/0.89
10 Distract	0.50/0.97	0.70/1.25	0.60/1.10

The means and standard deviations (SDs) of the number of micro-failure episodes and the number of occurrences of the nine categories of behaviors are shown. Number 1 in the “Behaviors” column refers to the average number of micro-failures that occurred. Numbers 2–10 refer to the average number of behaviors observed by category. “Select” and “Generate” refer to the two conditions being compared. The “Total” column on the far right refers to the overall mean by category, collapsing across conditions

### Relationship to learning

Our teachers were concerned that students’ general English and Science proficiency could influence their performance on the posttest since it required written responses and was in the domain of environmental science. To check for co-variance, we correlated students’ individual English and Science year-end exam scores with the posttests and found a positive correlation for both subject areas, *r* = 0.68, *P* < 0.01, and *r* = 0.42, *P* < 0.01, respectively. To compare learning with dyadic behaviors, we assessed remaining results at the dyad level by calculating the average of the partners within each dyad to produce dyadic posttest scores. With English and Science scores as covariates, an ANCOVA showed a significant effect of condition, *F*(3,16) = 8.89, *P* < 0.01, and we observed a moderate to large effect size, Cohen’s *d* = 0.77. Levene’s test showed equal variance between groups, *P* = 0.21. The Select group, *M* = 8.25, *s* = 1.40, outperformed the Generate group, *M* = 7.20, *s* = 1.34 (out of 12 points possible).

The behaviors in our failure episodes were not normally distributed, thus, we ran Kendall’s tau correlations on all behavior codes and posttest scores, collapsing across conditions. (See Table 3 below). There were no significant correlations between any single behaviors and scores. All correlations were small to moderate in magnitude (i.e., were not close to zero), except for the Distract variable, Kendall’s *τ* = −0.02. We observed a high variation in behaviors, which we expected due to the minimal guidance given for how to collaborate. In order to more broadly evaluate how students dealt with failure, we collapsed all behaviors that were positively correlated with scores into a one category and all behaviors that were negatively correlated with scores into another category. In this way, we could infer what was helpful or unhelpful for student learning while experiencing micro-failures. (The Distract variable was not included in either of these categories). We refer to the categories as Functional and Dysfunctional behaviors, respectively. Functional behaviors included numbers 2–5 from Table 2, while Dysfunctional behaviors included numbers 6–9. Because of high variability also in the frequency of behaviors across dyads, we converted the number of the Functional and Dysfunctional behaviors for each dyad into ratios over the total number of behaviors (2–10 from Table 2), in order to observe the relationships between the proportions of each type of behavior and posttest scores. (See Table 4 below). We used one-tailed Kendall’s tau tests to calculate the correlations, since each category was hypothesized to have a unidirectional relationship with posttest (i.e., Functional and positive, Dysfunctional and negative). There was a non-significant positive correlation between Functional behaviors and posttest scores, Kendall’s *τ* = 0.24, *P* = 0.09, and a significant negative correlation between Dysfunctional behaviors and posttest scores, Kendall’s *τ* = −0.31, *P* < 0.05.

**Table 3:** Correlations of dialogic behaviors and posttest scores

	Posttest scores	1	2	3	4	5	6	7	8	9	10
Posttest scores	1.00	0.21	0.13	0.24	0.34	0.26	−0.17	−0.09	−0.08	−0.19	−0.02
01 Num failures	–	1.00	0.31	0.48^a^	0.25	0.51^b^	0.05	0.34	−0.10	0.39^a^	0.31
02 Ques/exp peer	–	–	1.00	−0.04	0.35	0.07	−0.11	−0.19	−0.03	−0.12	−0.25
03 Ques/exp teacher	–	–	–	1.00	−0.16	0.76^b^	0.04	0.09	0.04	0.11	0.04
04 Argue/consens	–	–	–	–	1.00	0.0	−0.04	−0.20	−0.14	−0.18	−0.17
05 Call on teacher	–	–	–	–	–	1.00	−0.24	0.07	−0.24	0.10	0.18
06 Quick choice	–	–	–	–	–	–	1.00	0.13	0.40	0.26	−0.05
07 Argue/no consens	–	–	–	–	–	–	–	1.00	−0.03	0.83^b^	0.81^b^
08 Dom student	–	–	–	–	–	–	–	–	1.00	0.05	−0.21
09 Ignore	–	–	–	–	–	–	–	–	–	1.00	0.73^b^
10 Distract	–	–	–	–	–	–	–	–	–	–	1.00

^a^Correlation is significant at the 0.05 level (two-tailed)

^b^Correlation is significant at the 0.01 level (two-tailed)

We correlated the dyad posttest scores with the number of micro-failure episodes and the number of occurrences of each of the nine dialogic behaviors in each dyad. This analysis was done collapsing across the conditions for the whole data set. Kendall’s tau two-tailed tests were used to calculate correlations because the data was not normally distributed. Following across the first row within the table, note that posttest scores were not significantly correlated with the number of failures or any one behavior. However, also note that all correlations were small to moderate in magnitude, except for the Distract variable, which was near zero. The behaviors that showed small to moderate correlations with posttest scores were used in further analyses

**Table 4:** Correlations of behaviors by category and posttests

	1	2	3	4	5
1 Posttest score	1.00	0.28	−0.15	0.24	−0.31^a^
2 Num of functional behaviors	–	1.00	−0.14	0.69^b^	−0.63^b^
3 Num of dysfunctional behaviors	–	–	1.00	−0.62^b^	0.73^b^
4 Prop of functional	–	–	–	1.00	−0.85^b^
5 Prop of dysfunctional	–	–	–	–	1.00

^a^Correlation is significant at the 0.05 level (one-tailed)

^b^Correlation is significant at the 0.01 level (one-tailed)

The nine dialogic behaviors were collapsed into two categories. We included all behaviors that were positively correlated with posttest scores in a broader category named, “Functional,” while we included all behaviors that were negatively correlated with posttest scores in a broader category named, “Dysfunctional.” (See Table 3 for correlations of the nine behaviors and posttest scores). Correlations were calculated collapsing across conditions using Kendall’s tau due to non-normal distributions of the data. One-tailed tests were used since each broad category was hypothesized to have a unidirectional relationship to posttest scores (e.g., Functional/positive relationship, Dysfunctional/negative relationship). This table displays the correlations between the number of Functional and Dysfunctional behaviors in each dyad with posttest scores and the proportion of Functional and Dysfunctional behaviors with posttest scores. Owing to the high variation in behaviors, we calculated proportions by dividing the number of behavior occurrences in each broad category (Functional, Dysfunctional) by the total number of occurrences in order to observe a more sensitive measure of their relationships to posttest scores. Following across the first row within the table, note the significant correlation between the proportion of Dysfunctional behaviors and posttest scores

Owing to non-normal distributions, we used the non-parametric Mann–Whitney *U*-test to check for differences across conditions in the proportions of each type of behavior. There were no significant differences for Functional behaviors, *z* = −0.81, *p* = 0.46, or Dysfunctional behaviors, *z* = −0.81, *p* = 0.46. However, we note that the sample means aligned with the posttest results. Compared to the Generate group, the Select group used more Functional behaviors and fewer Dysfunctional behaviors on average, and showed better learning. See Table 5 for descriptive statistics.

**Table 5:** Means/SDs of behaviors by category and posttest

	Conditions	
Measure	Select	Generate
Posttest^a^	8.62/–	6.83/–
Behaviors^b^		
Functional		
Percentage	62.15/36.79	38.02/39.93
Mean rank	10.40	8.38
Dysfunctional		
Percentage	29.45/26.46	43.93/36.18
Mean rank	8.60	10.63

^a^Posttest means are adjusted for covariates

^b^Percentage of behaviors and Mann–Whitney mean ranks are shown

The means and standard deviations (SDs) of the prevalence of the broader categories of behaviors (Functional, Dysfunctional) are shown along with the adjusted means of posttest scores in each condition. The percentages of each type of behavior relative to the total number of occurrences and Mann–Whitney mean ranks are displayed. We used the Mann–Whitney *U-*test to calculate differences across conditions due to non-normal distributions. Posttest means were adjusted for covariates. “Select” and “Generate” refer to the two conditions being compared. Note that there was a higher percentage of Functional behaviors compared to Dysfunctional behaviors in the Select condition. The opposite holds true in the Generate condition, which shows a higher percentage of Dysfunctional behaviors

### Exploratory analysis of dyad behaviors

To further understand how dyads dealt with failure and speculate on possible differences across conditions, we first examined various groupings of dyads to look for patterns of behaviors and then provide a contrast of two cases illustrating the specific behaviors of a high-scoring dyad compared to a low-scoring dyad. Regarding general patterns, we found that the dyads from the Select group tended to engage in more questioning, explaining, and arguing to consensus during micro-failures compared to the Generate group. They also followed along the worksheet items, which seemed to keep them on task. They were more inclined to go in question order and partners tended to take turns sharing answers for each question. When one student skipped ahead, the partner often directed him/her back to the order of the questions. On the other hand, the Generate dyads tended to have a dominant speaker that ignored the partner’s responses and played random-chance games (e.g., rock, paper, scissors) to decide on item answers. These students also went off-task more frequently throughout the collaboration period compared to Select students. In some cases, students quickly listed answers right at the beginning, then discussed unrelated topics for the rest of their interactions. We did not observe other differences in the general patterns of interactions across conditions, but suggest the possibility that the Select group overall may have better managed their collaborative behaviors to stay on task. Further details about how we conducted these analyses can be found in the Supplemental Note 1 file. I next present the contrasting cases analysis.

We selected the highest-scoring dyad and the lowest-scoring dyad of the entire corpus in order to examine students’ dialogic behaviors and corresponding cognitive mechanisms around micro-failures at the extreme ends of the learning spectrum. The high-scoring dyad came from the Select condition and obtained a combined average of 85% on the posttest, while the low-scoring dyad from the Generate condition obtained a 33%. Being at the tail ends of the spectrum and from different conditions allowed us to infer some potential effects of the degree of open-endedness of the task design on students’ experiences of failure and their learning outcomes. The cases presented also support our quantitative analyses of Functional and Dysfunctional behaviors around failure and their relationships to learning.

**Fig. 2:**
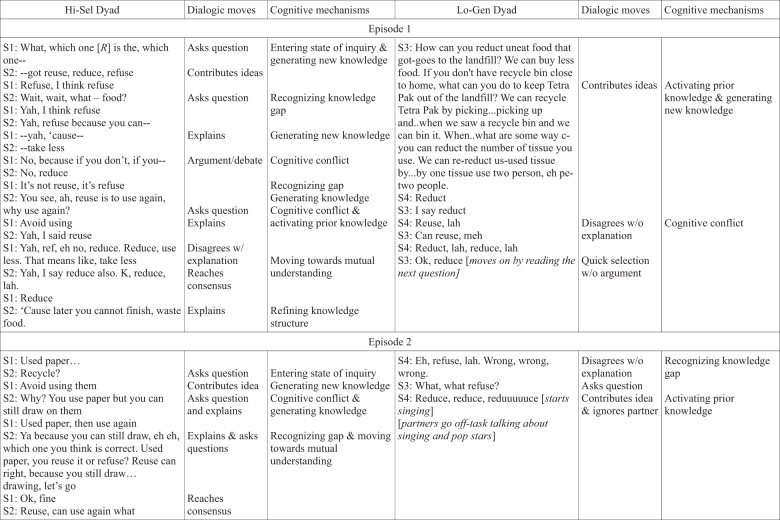
Excerpts contrasting high- and low-scoring dyads. The figure displays a side-by-side comparison of the dialogic utterances, moves (function of language), and corresponding cognitive mechanisms for the highest-scoring (Hi-Sel) dyad against the lowest-scoring (Lo-Gen) dyad relative to the whole data set. The Hi-Sel dyad was from the Select condition; the Lo-Gen dyad was from the Generate condition. Two episodes of micro-failures are shown for each dyad. S1 and S2 in the excerpts refer to the dialogic utterances of student 1 and student 2 of the Hi-Sel dyad. S3 and S4 refer to the utterances of student 3 and student 4 of the Lo-Gen dyad. Utterances were separated by speaker turns. The Hi-Sel dyad engaged in longer and more substantive interactions during micro-failures, which invoked a greater number and diversity of cognitive mechanisms. This comparison supports the finding that Select dyads interacted in ways that were helpful to learning while Generate dyads interacted in ways that hindered learning. This also mirrors the quantitative results showing a significant difference in learning outcomes, with students from the Select condition scoring higher on average than students from the Generate condition

Figure 2 illustrates the two dialogs, showing the comparison of their behaviors and the inferred corresponding cognitive mechanisms. The high-scoring dyad from the Select group (Hi-Sel) engaged in a substantive volley of conversation at their first micro-failure, which started with a question of which “R” best matched the worksheet item. The two partners showed evidence of reaching mutual understanding by taking short turns and finishing one another’s sentences. They also seemed to have entered a state of inquiry as they went back and forth, offering new knowledge to the discussion in the form of suggesting different “R’s,” providing explanations, and asking further questions. Finally, as they persisted in explaining and questioning, the discussion moved towards an argumentation type of dialog with each contributing different answers and explanations until they reached consensus. On the other hand, the low-scoring dyad from the Generate group (Lo-Gen) started with a dominant speaker rattling off her/his first three answers to the question items. The other partner interjected with a different answer, while the dominant speaker continued to state her/his answers. They engaged in some back-and-forth disagreement on the answer, but did not provide any explanations or additional knowledge to the discussion. There was not a sense that the two partners were working towards mutual understanding or consensus. The impasse ended with a quick selection of an “R” with no evidence of why this was chosen.

At the second micro-failure the Hi-Sel dyad discussed which “R” matched the item of ‘used paper.’’ This is a shorter interaction than the first, yet still showed evidence of entering an inquiry state through back-and-forth questioning and explaining, and recognition of knowledge gaps as ideas were contributed. They, again, appeared to move toward mutual understanding by discussing potential answers, however compared to their first interaction, the episode ended without further elaboration of their “R” selection. Yet, this was still a more substantive discussion than the Lo-Gen dyad. This is the only other micro-failure that the Lo-Gen dyad encountered during the full collaboration period. Their interaction around this impasse was very brief and happened in between the partners going off task (e.g., singing). There was a recognition of a knowledge gap at the start of the micro-failure, however the continued discussion included no explaining and showed no evidence of entering an inquiry state, moving towards mutual understanding, or refining knowledge structures. One partner asked a clarification question that was ignored. There was a quick disagreement and then a seemingly random selection. After the episode, they continued singing and going off task.

## Discussion

We identified several dialogic behaviors in which students engaged when they encountered micro-failures during collaboration. Those beneficial to learning were: questioning and explaining amongst each other, explaining after teacher prompts, engaging in argumentation and reaching consensus before moving on, and calling on a teacher for help. Our work showed that high scorers engaged in a higher proportion of these behaviors. In contrast, the following behaviors were shown to be disadvantageous to learning: making an immediate selection/response without coming to consensus, engaging in argumentation without consensus, having one partner dominate interactions, and ignoring an impasse and moving ahead. Low scorers engaged in a higher proportion of these behaviors. Becoming distracted during micro-failures did not have any conclusive effect on learning. The results of these specific behaviors and their relationships to learning are supported by other work in the collaborative learning literature.^29,35–37^ However, our work has empirically examined collaboration around micro-failures in the particular context of failure-based learning designs.

With regard to the mechanisms of learning that took place in our instructional contexts, we highlight the evidence of more helpful mechanisms occurring during collaboration after preparing in the highly structured activity. I offer some conjectures for the conditional differences on the learning results based on our exploratory analyses. The highest-scoring dyad was from the Select condition and seemed to enter into episodes of micro-failure in a state of inquiry. Through the activation and generation of knowledge, the partners recognized uncertainty and reached points of cognitive conflict. They engaged in questioning and explaining to address knowledge gaps and resolve inconsistencies, and continued to generate collective knowledge by contributing new ideas. The interaction moves gave the sense of trying to reach mutual understanding and consensus, where shifts of knowledge structures became possible. This pattern of dialog matches to Tawfik, Rong, and Choi’s cycle of working through micro-failures.^1^ The Select preparation task seemed to provide students with sufficient knowledge to access during collaboration, while collaborating allowed them to further differentiate the conceptual features of the “R’s” by interacting in ways that facilitated learning (e.g., explanation, elaboration, etc.). We also saw evidence of knowledge assembly during collaboration through back-and-forth contributions of ideas and questions for understanding.

What we observed in our low-scoring Generate dyad supported the significant negative correlation between Dysfunctional behaviors and learning. There were a few instances of gap recognition and cognitive conflict, but the partners did not engage in dialogic moves that addressed these. There was little evidence of explaining, elaborating, or providing justifications for item answers, and they tended to ignore one another’s ideas. Additionally the analyses of different groupings of dyads across the conditions showed that over half of the dyads from the Select group engaged in at least one instance of argumentation to reach consensus while no dyads from the Generate group engaged in this behavior. Considering the sole interaction of reaching consensus as a form of assembling knowledge, the Select dyads had an advantage. Both groups would have had relatively equal chances to assemble knowledge throughout the direct instruction, however, the Generate dyads missed the opportunity for knowledge assembly during collaboration. This may have left them with knowledge structures that were more faulty or naive at the start of the direct instruction. This, coupled with our impression that Generate dyads went on-and-off task relatively frequently, may have disrupted the chance for improving understanding after a micro-failure. Below I speculate on why our students from the Generate condition might have behaved differently.

Our sample came from a younger population than those of prior studies that have examined outcomes from failure-based learning designs. Our students were in their fourth year of primary school, from age 9–10, rather than at secondary or tertiary levels of education. There are studies that support a potential moderating effect of age/developmental stage on learning from ill-structured problem solving, where there is opportunity to encounter failure.^38,39^ This may be due to young children having more difficulty in self-regulating behavior during challenging learning tasks.^40^ Experiencing failure, dealing with a highly open-ended task, plus engaging in collaboration requires simultaneous emotional, cognitive, and social regulation, and our Generate students may not have been able to cope with such a high demand of self-regulation. This could be why they seemed to veer off task more regularly, perhaps using these “breaks” as a coping mechanism. In addition, we also note Mende, Proske, Korndle, and Narciss’s study^28^ that found a moderating effect of prior knowledge on ill-structured collaborative tasks, showing that higher prior knowledge groups performed better with an ill-structured task while lower prior knowledge groups performed better with a well-structured task. Although their study was done on older students, age/development can also be perceived in terms of prior knowledge, or in other words, prior experience. Younger children are less knowledgeable and less experienced, thus, our Generate students may have been hindered by the lack of structure of the task.

From a cognitive load perspective, it is possible that students in the Generate group experienced higher cognitive load and were, consequently, incapable of engaging in more effective dialogic behaviors. Questioning and explaining, as well as engaging in argumentation through to consensus are interactions in which it is particularly difficult to persevere.^37,41^ The increased degree of open-endedness in the Generate task may have reduced the cognitive capacity for our students to persist through these difficult interactions by the time they entered the collaboration phase. It is easier to ignore disagreement, quickly make decisions and choices, and not persist to reach consensus, which are behaviors that tend to be indicators of cognitive overload in collaborative learning and were found to be more frequent in the Generate group’s interactions. Relatedly, since our design separated the exploration task to first have an individual phase of activity, it is possible that the Generate students utilized most of their cognitive resources at that time. When they reached the collaboration phase and especially when they encountered micro-failures, they may have had the capacity only to make quick decisions, let a partner dominate, or simply randomly pick answers and move on. On the other hand, the structure of the task for the Select group could have provided a better opportunity for germane cognitive load, while reducing extraneous load.^42,43^ The need to make decisions on four choices (e.g., the four Rs) may have still been complex enough for the students to substantively engage in sufficient “thinking work,” but did not overwhelm them with too much open-endedness, thus leaving more cognitive resources for effective collaboration.

Work from the argumentation literature has found that there can be a dip in learning immediately following an instructional intervention.^44^ It is possible that the Generative group would perform better in a delayed posttest, which more closely aligns to the productive failure work showing benefit of ill-structured over well-structured problem solving.^9^ However, we do not believe this to be a strong conjecture, considering the evidence of our Select dyads utilizing a greater proportion of Functional behaviors and a lower proportion of Dysfunctional behaviors compared to the Generate dyads. Based on the poorer performance of the Generate group, one interpretation could be that failures simply were not productive in our context, i.e., the productive failure hypothesis was not true here. I interpret this differently. The students from our more well-structured condition experienced productive micro-failures and encountered more failures on average compared to the Generate group (although this was not a significant difference). Thus, it would be misleading to conclude that we did not see any learning benefits from failure, but rather that the Select group better evidenced it.

One major limitation of this work is the small sample size, which was reduced due to lack of participant consent to have their data analyzed. With twenty dyads, especially across two conditions, we caution against any strong claims. Another limitation is the lack of generalizability to other domains. This work was done on a human behavioral aspect of environmental education (how to best use the “R’s”) and should be interpreted within that scope. The short timespan of the learning activity also limits the generalizability of the work in that additional learning benefits of failure could be studied more comprehensively from longitudinal studies. However, our work allowed for understanding of student learning in natural classroom settings as they occur within the context of a lesson. Thus, our contribution falls within the broader context of learning. Next, we did not use a control condition where we could test the effect of the individual preparation without collaboration, which may have provided further insights into how much learning occurred specifically in the collaboration phase as separate from the instructional phase following. This would have allowed us to infer more about the influence of interactive behaviors that occurred during micro-failures on learning outcomes. Finally, to date, research has not determined if failure-based learning approaches benefit elementary students in any domain. Empirical studies are needed to test the effectiveness of productive kinds of failure for this age group. Experimental research that could isolate subject area and age, as well as better control for external factors (as opposed to our work that was done in-vivo), would satisfy this gap and points to an important direction for future work.

There are a few potential confounding factors of our work. One is in level of teacher intervention compared to other work that has been done in failure-based instructional contexts. The teachers and facilitators had the freedom to help students as they felt was needed, which mostly took the form of generic prompting such as:

- Can you think of examples when you [recycled, reused an item, etc.]? Share and ask your partner what s/he thinks. I will check on you in a few minutes.
- You both think that [refuse] is the best one? Do you know why? It is ok, slowly. You can take your time.

However, there were a few instances where teachers gave content-based feedback during collaboration, which would not be encouraged during a productive failure type of exploration. This was particularly noticeable in two dyads from the Select group. This was unavoidable in the classroom context. Rather than this negating the result of the Select group performing better, I offer an alternative perspective. If teacher intervention would generally be considered a hindrance to learning from failure, then it should have disadvantaged the Select group. Yet, we observed that these dyads interacted productively within micro-failure experiences and showed better learning. The other confound comes with our contrasting cases analysis. The highest-scoring dyad happened to be from the Select condition and the lowest from the Generate condition. Thus, I only speculate on potential influences of task open-endedness on dialogic moves and am careful not to make claims on causal effects based on this analysis. However, I note that the additional analyses of the various groupings of dyads, as well as the quantitative findings across conditions align with the results from the contrasting cases. All findings support a benefit of the Select task.

With respect to the limitations, which were partly due to a lack of experimental control, I consider a compromise of internal validity a worthwhile tradeoff for high external validity. Our work was done in real classrooms during normal school hours, within the regular context that teachers and students operated. Using a quasi-experimental design allowed us to examine different instructional activities with minimal disruption to the norms and dynamics of each class. Furthermore, we did not impose strict guidelines for how to work on the activities or how to collaborate, leaving room for students to engage naturally in the work. Thus, despite the limitations, there is strength in the findings being generalized to similar authentic classroom settings.

Lastly, I offer research questions for future work. First, regarding our PFC design, is failure during the individual preparation phase a necessary component for later learning, and relatedly, how could micro-failures be identified at this phase? Then, what effects would failing at this phase have on collaborative behaviors and learning outcomes? Second, what kinds of content-based failures lead to functional or dysfunctional collaborative behaviors? Studies designed specifically to induce failure based on canonical forms of concepts could start to address such questions. Third, what is the relationship between number of micro-failures and learning outcomes? Our work did not find a significant correlation, but our sample size was limited. Additional studies would be needed. Finally, how do individual partners benefit from collaboration when they encounter micro-failures? This would involve a different focus that examined the individual’s contributions to a dialog. Such a next step could further inform the process of collaboratively learning around failure.

## Methods

### Ethics statement

Informed written consent from all student participants and their parents were obtained according to the procedures approved by the Nanyang Technological University’s Institutional Review Board (IRB-2015-06-034-02).

### Participants

The discourse analyzed in the current work came from two low-performing fourth-grade classes in a Singaporean primary school. (Students are typically placed into “ability” based classes according to performance on year-end exams in early primary school). The sample consisted of all student pairs who provided consent to have their data analyzed, *n* = 40 students, 20 per condition. The author of this paper taught the lessons for both classes, with assistance from the respective homeroom teachers and two research team members. All adult participants who assisted were briefed on addressing students through generic prompting during the preparation and collaboration phases. It was emphasized that teachers should not “give away” any answers to students, nor complete any portions of the work for the students. However, they were told that they could help the students to stay on task and manage their behaviors if needed (e.g., redirecting off-task discussion, not talking, or seeing a dominating partner during collaboration).

### Operationalizing the dialogs

**Fig. 3:**
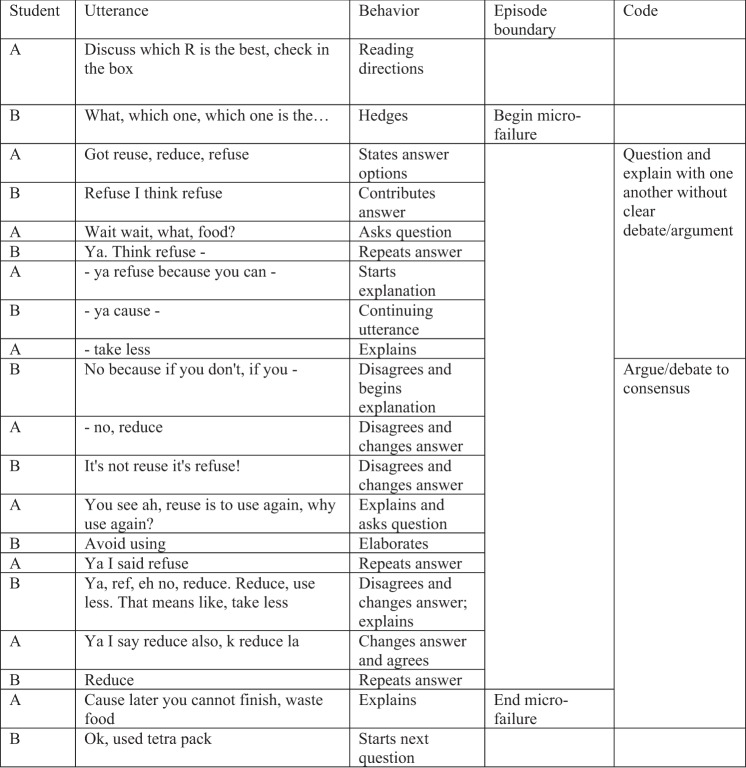
Example illustrating development of codes. The figure shows an episode of a micro-failure. The episode begins with the start of a question item where the students are hedging. Hedging indicated entering into a micro-failure. Here students A and B are discussing which “R” goes best with the item of leftover food. The episode ends when the students start to discuss a new item. Utterances were separated by speaker turns. In order to develop our coding scheme, we recorded the functional behavior of each utterance, as shown in column 3. Related utterances were then collapsed into broader dialogic categories, as shown in the far right column. In this example, the students initially engaged in the broader categories of (**a**) questioning and explaining without debate, and (**b**) arguing to consensus

We analyzed verbatim transcripts of the student dialogs. Micro-failures were operationalized by moments when students overtly displayed awareness of an impasse in their collaborative discussions. We singled out each occurrence of a micro-failure as an “episode”, which consisted of a segment of discussion where one or more actions occurred: verbalization of awareness of uncertainty or gaps in knowledge, disagreement, questioning one another or a teacher, long pauses, or stutters/hedges, which follows theories related to cognitive linguistics and impasse-driven learning.^45,46^ We segmented episodes by a switch from peer dialog to talking with a teacher or if students shifted to a different content topic, using Chi’s guide for quantifying verbal data.^47^ Within failure episodes, we identified specific student behaviors based on the function of the language in a bottom-up fashion according to Chi and Menekse’s work on coding for interactive actions.^48^ We collapsed the behaviors into nine categories that comprised our scheme to code all failure episodes. Since a failure episode could include several utterances, it was possible for more than one category to occur in a single episode. An example showing this process is included in Fig. 3. It was coded as a single episode that began with a hedge and ended with a switch in content topic (e.g., from food waste to tetra pack). In the first half of the example, contributing answers, repeating statements, explaining, and asking questions were collapsed into the “question and explain with one another without clear debate/argument” code. In the latter half, disagreement, explaining, changing answers, questioning, repeating answers, and coming to agreement were collapsed into the “argue/debate to consensus” code.

### Prior study procedures and lesson materials

Since our analysis of discourse came from two different conditions of our prior study, I further describe the learning tasks here. We conducted a quasi-experimental study investigating PFC by randomly assigning each of two intact classes to a condition: Select vs. Generate. To avoid teacher effects, the same teacher facilitated the lessons in both conditions. Teacher-led instruction and time on task were held constant across the two conditions. After the lesson, students were given a posttest where they individually engaged in a complex problem-solving task around the same topics. Specifics are provided below.

#### Introduction of problem

The teacher presented some problems of overproduction of waste and introduced the four R’s in a 20-min whole-class presentation.

1. Preparation activity. Students then individually engaged in the preparation phase for 30 min. They were asked to complete a worksheet alone, but could ask a teacher for clarification of instructions. The conditional manipulation was done during this phase. The Select worksheet was made up of ten question items. Each item showed a picture of a different piece of trash (e.g., leftover food, used paper, soda can). For each picture, students were instructed to circle the R that best fit each item. The Generate worksheet included the same ten picture items. However, each item was phrased as an open-ended question that instructed students to generate examples of what to do with the item based on a specific R action. For instance, “How can you reduce uneaten food that goes to the landfill?” Students were told to generate as many examples as they could for each question item in the time allotted.
2. Collaboration activity. Students were paired within condition, either self-selected or with help from teachers as was typical in their daily classroom activities. They worked on a joint worksheet that mirrored the Select preparation worksheet for 20 min. Students could use their individual preparation work as a resource. The instructions for collaboration were the same across groups: to discuss each item, to come to consensus on “the best R,” and finally circle an agreed upon answer. We expected the content produced in the preparation task to drive the discussions. In other words, students in the Select condition would have had their selected R and the item prompts available for discussion, while the Generate students would have had their generated ideas for a given R in each item prompt. If students could not agree upon an answer, they were asked to make a note of their disagreement on the worksheet.
3. Direct instruction. The teacher provided a 10-min consolidation presentation that explained the four R’s in detail and how each R helped decrease waste to the waste management system. After the presentation, the students watched a 10-min video about the importance of being environmentally friendly.

#### Posttest problem

The day following the lesson, the students were given 30 min to individually write down a solution to the problem below:

The growing waste in Singapore is a complex problem. There are many parts of the problem, and many solutions that can help to fix the problem. Write a BIG solution to help! This can include many smaller solutions. Or, it can be one BIG idea that fixes many parts of the problem.

There was space to write up to a half-page. The test included a basic information sheet on the four R’s with these points:

- *Different actions can be taken with different kinds of waste*.
- *Recycling allows materials to be remade into new products*.
- *The 4R’s help prevent the growing waste to landfills*.
- *Buying and using only what you need prevents growing waste*.

Ninety minutes comprised the lesson and 30 min were devoted to the posttest, totaling 2 h for the study implementation. Further details on the teacher-led presentations, activity worksheets, and posttest can be viewed in the Supplemental Methods file.

### Quantitative analysis of data

We used the students’ posttest responses from the study described above for the measure of learning in the current work. They were scored according to how novel, comprehensive, integrated, and accurate they were in content. (The scoring rubric is included in the Supplemental Note 2 file). Two raters scored all responses, showing good consistency, *ICC*(2,2) = 0.83, *P* < 0.01. ANCOVA was used to determine differences across conditions on the posttest scores and Pearson’s *r* was used to indicate the relationships between covariates and posttest scores. Two-tailed and one-tailed Kendall’s tau correlations were used to determine relationships between discourse variables and posttest scores, and Mann–Whitney *U*-tests were used to determine differences across conditions on the discourse variables. Information regarding analyses is also included in the Results section.

## Supplementary information

Supplemental Materials

## Data Availability

The data that support the findings of this study are available from the corresponding author upon reasonable request.

## Supplementary information

**Supplementary information** accompanies the paper on the *npj Science of Learning* website (10.1038/s41539-019-0045-1).
