# Supplementary material for: What students do when encountering failure in collaborative tasks
Source: NPJ Sci Learn. 2019 May 31;4:6. doi: 10.1038/s41539-019-0045-1 (PMC6544644; doi:10.1038/s41539-019-0045-1)
Supplement: Supplementary file 1 — Supplemental Materials [file 41539_2019_45_MOESM1_ESM.pdf]

Supplemental Methods

Additional information on instruction from prior study:

Learning materials

Teacher presentation. The introductory presentation was in the form of a PowerPoint. It included information about the environmental problems that arise from the overproduction of waste in densely populated urban areas. For example, harm to plants and animals in the environment and increasing likelihood of people falling ill due to toxins. Specifically, it introduced the main problem of the increasing production of waste in Singapore and the lack of capacity for Singapore's existing waste management system to handle this. Finally, it introduced the "four R's" of Refuse, Reuse, Reduce, and Recycle via brief descriptions.

Teacher consolidation lesson. First, a presentation also in the form of a PowerPoint covered explicit information on how each R helps to decrease waste in the environment. For example, Reusing a water bottle prevents the plastic or glass from entering the waste system and Recycling a metal can allows for the metal to be made into another product, rather than be added to landfills. After the presentation, the students watched a video about the importance of engaging in environmentally friendly actions. The video was animated with bear characters and focused on the use of tote bags.*

*The video may be viewed on Cartoon Network, We Bare Bears, Season 1, episode 19.

Supplemental Note 1

Groupings of dyads for comparison analyses: First, we compared the top three and bottom three scorers of all dyads. The top scorers came from the Select group. Their posttest scores were 10.25, 9.00, and 8.75. The instances in which they engaged in Functional and Dysfunctional (F:D) behaviors were 7:0, 9:1, and 3:2, respectively. All three dyads engaged in questioning and explaining with a partner and arguing to consensus during micro-failures. Dyad 2 additionally engaged in explaining after teacher prompts and initiating teacher help. Sample excerpts from Dyads 1 and 2 illustrate the different interactive behaviors of the students (see below). For instance, in Dyad 1, both students contribute ideas and explanations and question each other as uncertainty or disagreement arises. In one episode (A), they start with the idea of “recycle,” but then build upon the “reuse” concept with explanations and clarifications to reach agreement. In another episode (B), they initially build upon the “refuse” idea, then reach a disagreement when a partner introduces “reuse” and presses the other for an explanation. When the student explains and elaborates on why “refuse” is the better answer, the partner agrees. Dyad 2 shows much more uncertainty in their interactions and their collaboration includes some teacher prompting. In an episode (A) where a teacher prompts for responses, a student is unable to explain why s/he and the partner chose the “reuse” concept. The students in this episode are not able to generate an explanation in the moment and the teacher instructs them to move ahead once they agree, which seems to cut this episode short. However, as the students continue and discuss the next question (Episode B), they engage in a much more substantive dialogue where they offer answers and ideas with explanations and questioning. One student displays uncertainty about his/her own understanding about the “recycle” concept, with hedging and questioning, but also shows persistence by restating that “recycle” is the answer. The partner then disagrees and explains why “reuse” is a better answer, to which the student finally agrees. To summarize, although there is variety in how the top scoring dyads behaved, there seems to be a common pattern of working to reach consensus. This distinguished them from the bottom three scorers, on which we elaborate below.

The bottom three scorers included two Generate dyads and one Select dyad. Their posttest scores were 4.00, 5.00, and 6.75. The instances in which they engaged in F:D behaviors were 0:2, 2:6, and 1:7, respectively. During micro-failures, Dyad 4 only had instances of a partner dominating the interaction to answer questions, Dyad 5 had instances of engaging in argumentation without consensus and ignoring impasses, and Dyad 6 engaged in all four kinds of Dysfunctional behaviors at different points. Excerpts from Dyads 4 and 5 are used to illustrate the behaviors (see Figure 2). In Dyad 4, the two failure episodes are brief and end abruptly with one student deciding on the answer to the items. The students have disagreement by merely stating alternative answers, but do not engage in any explanation or elaboration on their ideas. They essentially state answers, and then one of them changes the answer without further discussion. There is no indication of working together to reach a consensus. For Dyad 5, in the first episode (A), the students take turns to state their answers and before any discussion ensues, one suggests a random-chance game (scissors paper stone) to make a decision. The partner tries to reengage the student by saying, “Let’s talk about

it” first. They subsequently take turns explaining and elaborating, but then the student suddenly says, “Let’s go with your [i.e., the partner’s] answer.” In the next episode (B), they state and repeat answers, one provides an explanation for an answer but then quickly starts the random-chance game. The episode ends with the “winner” announcing that his/her answer is chosen, ignoring the partner’s contribution and ending the episode. Thus, as compared to the high scoring dyads, there is little evidence of these dyads exerting effort in their discussion to negotiate meaning or understanding towards consensus. Rather, a student quickly made decisions and moved forward in the task, ignoring or dominating his/her partner.

To determine if these high and low scoring dyads were generally representative of the conditions from which they came, we also looked at the bottom three Select and top three Generate dyads. The Select posttest and F:D scores were 5.00, 7.00, 8.00, and 2:6, 3:7, 3:0, respectively. The Generate scores were 8.75, 8.50, 8.25, and 1:7, 9:0, 5:2. For the bottom Select dyads, two had an instance of coming to consensus. For the Generate group, none had instances of coming to consensus. In fact, across the entire corpus, we note that in the Select group, six of 10 dyads had at least one instance of coming to consensus while none of the dyads in the Generate group showed this behavior during failure episodes. Since the failure episodes did not comprise the entire collaborative period, we looked at the entire dialogues of the 12 dyads (Select – 3 high, 3 low; Generate – 3 high, 3 low) to see whether any other patterns emerged. The data did not show clear patterns, but generally pointed to a few similarities within conditions. In the Select group overall, the students followed along the worksheet questions which seemed to keep them on task. In five of the six dyads, the students went in question order and took turns sharing answers in each question. When one student skipped ahead, the partner often directed him/her back to the order of the questions. In the Generate group, two of the high scorers went mostly in question order, but also went off-task every one to two questions. The other high scorer started with the last question, stated uncertainty about what to do, had generic prompting from a teacher, and then went on and off-task talking over only three of the 10 items. Two of the low scorers quickly listed several answers right at the beginning in turns, then went on and off task throughout the rest of their interactions.

High-scoring Dyads

Dyad 1	Categories/behaviors	Dyad 2	Categories/behaviors
<u>Episode A</u><br>S1: Used paper<br>S2: Recycle?<br>S1: Avoid using them<br>S2: Why? You use paper, but you can still draw on them.<br>S1: Used paper, then use again.<br>S2: Ya because you can still draw. Which one do you think is correct? Used paper. You reuse it or refuse? Reuse can right, because you still draw.<br>S1: Ok, fine.<br>S2: Reuse.<br><br><u>Episode B</u><br>S1: K. Plastic straw waste. We should refuse to use it<br>S2: Because if we use it a lot…<br>S1: If we use it a lot.. We just throw away, but if you use met, metal?<br>S2: You never use metal.<br>Plastic. We should reuse plastic straw waste, plastic straw. Ya what! Why refuse? There's no straw what, paper straw.<br>S1: If you use plastic straws, later you will, you will waste it and you will go the same thing to the, ash place and pollute the air. You can just pour the water from anything and pour into your cup and drink it.<br>S2: Oh great great! Great! *[inaudible]*	**Explain & question (partner)**:<br>Reads item.<br>States answer as question.<br>States idea.<br>Asks question for explanation.<br><br>States idea.<br>Agrees and elaborates.<br>Asks question for clarification.<br>States answer w/ explanation.<br><br>Agrees.<br><br>**Argue to consensus**:<br>States idea.<br><br>Starts explanation.<br>Completes explanation.<br>Asks question offering idea.<br>Disagrees.<br>Disagrees/states alternative idea.<br>Asks question for explanation.<br><br><br>Explains & elaborates<br><br><br><br>Agrees.	<u>Episode A</u><br>T: You all think that reuse is the best way to do with plastic bag? Both of you?<br>S1: Uh huh.<br>T: Ok, do you know why?<br>S1: Er…<br>T: Why do you all think it's reuse?<br>S1: Because I… we don't know what to say.<br>T: It's ok. Slowly, take your time.<br>S1: Er… So that you can reuse it into like other, you can reuse plastic bag, reuse er… other what? What is that?<br>T: No need to keep taking more? Ok. As long as you both agree.<br><br><u>Episode B (immediately following)</u><br>S2: Er… my one is recycle. You? (57)<br>S1: Why you think it's recycle? I want reuse, because we can put the pen refill in the pen, right? So you don't need to throwing all the pen. You just need to buy the pen refill. So you don't need [to buy] this pen. Why do you choose recycle? … Is it because you think you can change into this one?<br>S2: Because… inside is the pen right? Then… er, no. No this one is pen and paper, right? Then somebody… eh, like this. No. …<br>[*some hedging for both students*]<br>S2: So, I choose recycle.<br>S1: You can reuse, you can keep reusing, like using the pen refiller. How about you? … You think it's reused? Reused? Reused?<br>S2: Er… Reuse.	**Explain & question (teacher)**:<br><br>Teacher prompt.<br>Hedges.<br>Teacher prompt.<br>Hedges.<br>Teacher prompt.<br><br>States uncertainty.<br><br><br>Starts explanation.<br>Asks question when uncertain.<br>Teacher instruction.<br><br><br>**Argues to consensus**:<br>States answer & questions partner.<br>Asks question for explanation.<br><br>Restates answer & explains.<br><br>Elaborates.<br>Asks question for explanation.<br>States idea as question.<br><br>Starts explanation & states uncertainty.<br>Questions self.<br>Hedges.<br><br>Restates answer.<br>Disagrees.<br>Offers explanation.<br>Questions partner.<br><br>Changes answer and agrees.

Low-scoring Dyads

Dyad 4	Categories/behaviors	Dyad 5	Categories/behaviors
Episode A	**Quick choice-dominate partner**:	Episode A	**Argues without consensus**:
S1: What are some ways you can reduce the	Reads item.	S1: [*re: tissues*] In my opinion, it's	States answer &
number of tissue you use. We can reduce used	States answer.	reduce. What about you?	questions partner.
tissue by... by one tissue use two person, eh		S2: Recycle.	Disagrees/states alternative answer.
pe-two people.		S1: Let's scissors, paper, stone. Scissors	Suggests random-chance game
S2: Reduce.	(Wrongly) corrects partner.	paper stone!	to choose answer.
S1: I [said] reduce.	Repeats answer.	S2: Let's talk about it, then scissors	
S2: Reuse.	States an alternative answer.	paper stone. Let's talk about is.	Task coordination.
S1: Can[not] reuse, meh.	Disagrees.	S1: Reduce because we must use the	Repeats answer.
S2: Reduce, reduce.	Changes answer.	tissue so we don't waste [them]. If we	Explains & elaborates.
S1: Ok, reduce.	Repeats answer.	waste [them], it goes to the landfill and	
		the year of 2035, it will be full and our	
Episode B		lands will be rubbish!	
S2: [*re: food item*] Refuse! Refuse, bro.	States answer.	S2: Mine is recycle because we can	Repeats answer.
S1: Bro, bro.		recycle paper! Paper is tissue, it's a type	Explains & elaborates.
S2: Eh, this one you can copy over here.	Task coordination.	of paper. Yes, but it's a type of paper.	
… Eh, refuse wrong, wrong wrong!	Disagrees.	Let's go with your answer. Let's go, now	Changes answer & ends episode.
S1: What? What refuse?	Asks question for clarification.	it's your turn to write in the book.	
S2: Reduce, reduce, reduce.	Changes answer		
[*goes off-task and starts singing*]	& ends episode.	Episode B	**Ignores and moves forward**:
		S1: Next question, 8. [*re: straws*] In my	States answer &
		opinion, it's recycle. What about yours?	questions partner.
		S2: Reuse!	States answer.
		S1: Reuse.	Repeats partner's answer.
		S2: Reuse because, wait. Because I can	Repeats answer.
		ruse straws if I don't use straws to drink	Explains & elaborates.
		water and a packet of milo. Scissors	Uses random-chance game.
		paper stone! Scissors paper stone! Yes.	Wins game for answer.
		S1: Nooo!	Expresses disappointment at loss.
		S2: Yes, yes, yes! I'm the winner!	Clarifies win for answer.

Supplemental Methods
Select Worksheet

Name________________________ Individual - SS

Make LESS Waste!

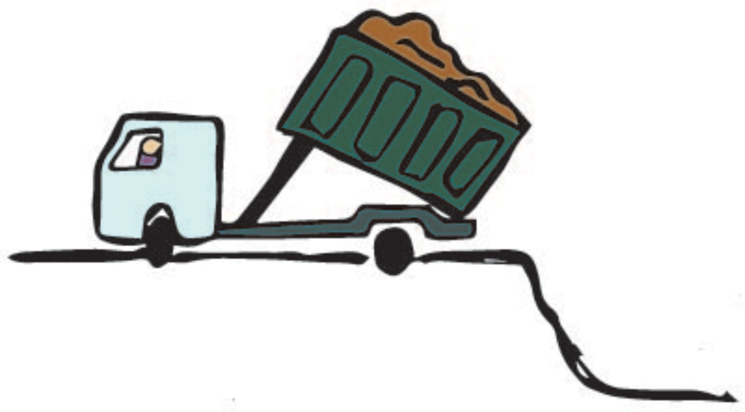

- Singapore's landfill may be full by 2035.
- We all need to help prevent waste going in the landfill.
- We can do this with the 4 R's – **REUSE! REDUCE! REFUSE! RECYCLE!**

Instructions

- Look at the 10 items. Read the sentence next to each item.
- Decide which R to use so that the item does not enter the landfill.
- Circle the R that fits in the blank.

	**Circle the R that fits best.**
1. 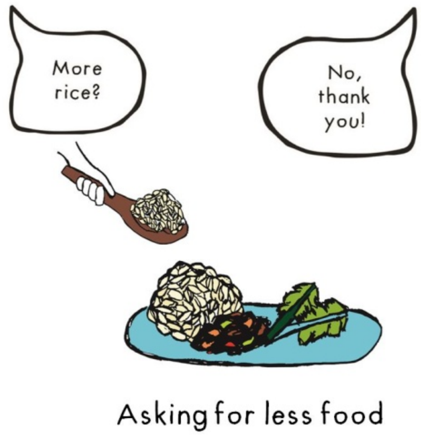	By choosing NOT to take more rice, you _______ food going to the landfill.<br>**Reuse**<br>**Reduce**<br>**Refuse**<br>**Recycle**

	**Circle the R that fits best.**
2.<br>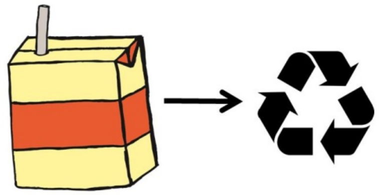<br>Used tetra pak	You can_______ your empty tetra pak instead of throw it away.<br><br>**Reuse**<br><br>**Reduce**<br><br>**Refuse**<br><br>**Recycle**
3.<br>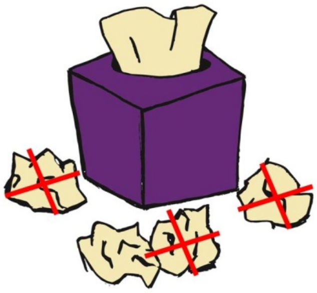<br>Used tissues	You _______ tissues to throw away by using less to wipe your hands.<br><br>**Reuse**<br><br>**Reduce**<br><br>**Refuse**<br><br>**Recycle**
4.<br>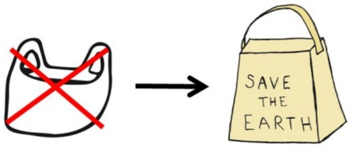<br>Plastic bags	When you bring a tote bag to the store, you can _______ plastic bags.<br><br>**Reuse**<br><br>**Reduce**<br><br>**Refuse**<br><br>**Recycle**

Circle the R that fits best.	
5.<br>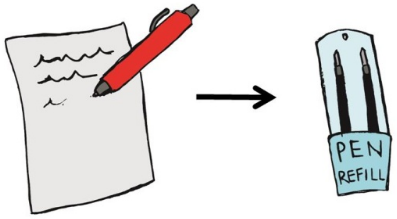<br>Used pen	When your pen runs out of ink, you can ________ it by replacing the ink.<br><br>**Reuse**<br><br>**Reduce**<br><br>**Refuse**<br><br>**Recycle**
6.<br>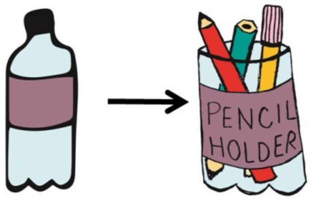<br>Plastic bottle	You _______ an empty water bottle by making into a pencil holder.<br><br>**Reuse**<br><br>**Reduce**<br><br>**Refuse**<br><br>**Recycle**
7.<br>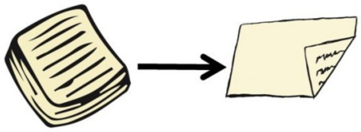<br>Used paper	You _______ paper when you use the back to write on.<br><br>**Reuse**<br><br>**Reduce**<br><br>**Refuse**<br><br>**Recycle**

	Circle the R that fits best.
8.<br>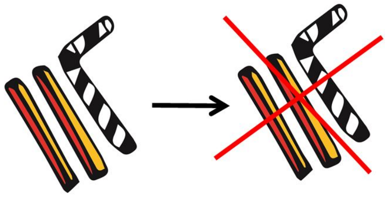<br>Plastic straws	When you drink directly from a can of soda, you can ______ plastic straws.<br><br>**Reuse**<br><br>**Reduce**<br><br>**Refuse**<br><br>**Recycle**
9.<br>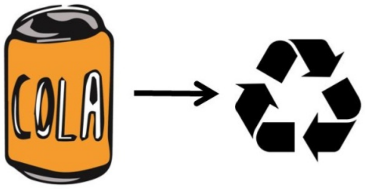<br>Used can	You can_______ your empty soda can instead of throw it away.<br><br>**Reuse**<br><br>**Reduce**<br><br>**Refuse**<br><br>**Recycle**
10.<br>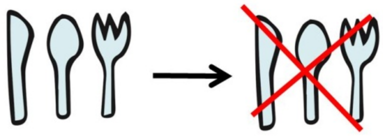<br>Plastic cutleries	When you use metal cutleries at the canteen, you can _______ plastic.<br><br>**Reuse**<br><br>**Reduce**<br><br>**Refuse**<br><br>**Recycle**

Name______________________ Individual - GS

Make LESS Waste!

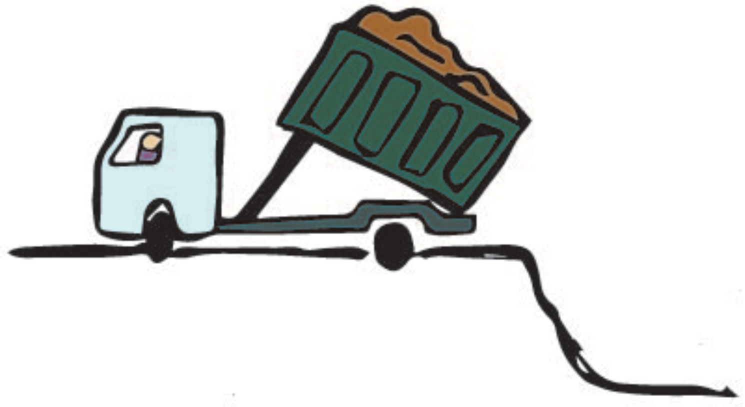

- Singapore's landfill may be full by 2035.
- We all need to help prevent waste going in the landfill.
- We can do this with the 4 R's – **REUSE! REDUCE! REFUSE! RECYCLE!**

<u>Instructions</u>

- Look at the 10 items. Read the question next to each item.
- Think about what you can do so the item does not enter the landfill.
- Write your answer in the blank.

	**Write your ideas below.**
1. 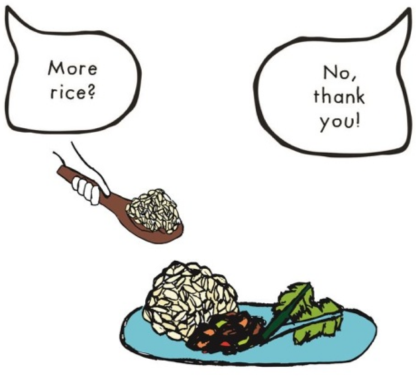 Asking for less food	How can you **<u>reduce</u>** uneaten food that goes to the landfill?

	Write your ideas below.
2.<br>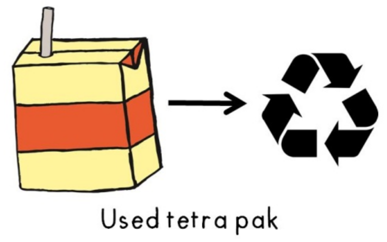	If you don’t have **recycle** bins close to home, what can you do to keep tetra paks out of the landfill?
3.<br>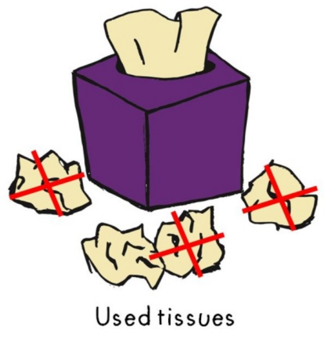	What are some ways you can **reduce** the number of tissues you use?
4.<br>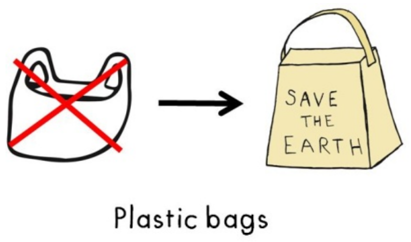	If you **refuse** plastic bags at the store, what are other ways to get your items home?

	**Circle the R that fits best.**
5. 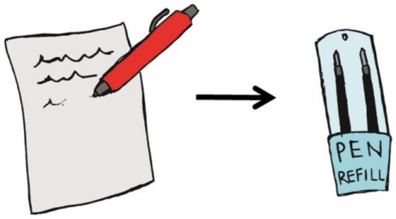 Used pen	If your pen runs out of ink, what could you do to **<u>reuse</u>** it?
6. 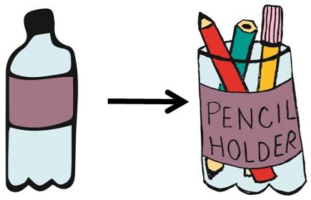 Plastic bottle	How could you **<u>reuse</u>** an empty water bottle?
7. 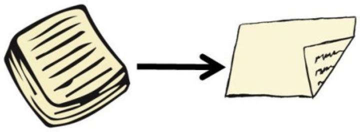 Used paper	How could you **<u>reuse</u>** paper that is marked on only 1 side?

	Circle the R that fits best.
8.<br>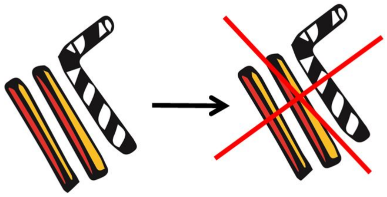<br>Plastic straws	Plastic straws can be **refused**. What can you do instead of using a straw?
9.<br>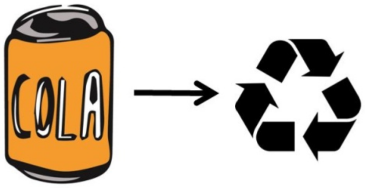<br>Used can	Your empty can is sticky from the soda you were drinking. What can you do to be sure it gets **recycled**?
10.<br>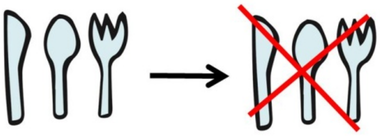<br>Plastic cutleries	You go to the hawker center to eat dinner. If you **refuse** plastic cutleries, what can you use?

Name: ________________________ Individual Post – L3

Make LESS Waste!

<u>Review of Lessons</u>

You have been learning about **Singapore's growing waste problem** and how to prevent waste from going to the landfill. Some main points include:

- Different actions can be taken with different kinds of waste.

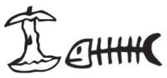

Food

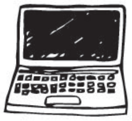

E-waste

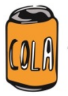

Metal

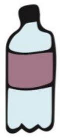

Plastic

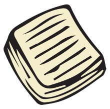

Paper

- Recycling allows materials to be remade into new products:
  - keeps the used items out of landfills
  - saves new resources from being used

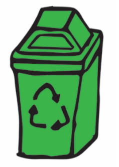

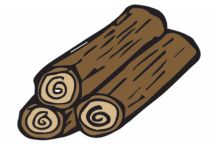

- The 4 R's help prevent the growing waste to landfills:

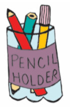

  - **<u>Reuse</u>** an item as many time as possible
  - **<u>Reduce</u>** by taking or using less
  - **<u>Refuse</u>** by finding other ways
  - **<u>Recycle</u>** metals, papers, and plastics

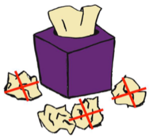

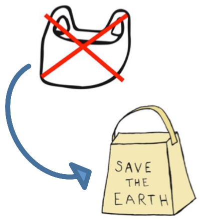

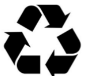

- Buying and using **only what you need** prevents growing waste!

Finding a Solution

The growing waste in Singapore is a **complex problem**. There are many parts of the problem, and many solutions that can help to fix the problem.

Write a BIG solution to help! This can include many smaller solutions. Or, it can be one BIG idea that fixes many parts of the problem.

____

____

____

____

____

____

____

____

____

____

____

____

____

____

Helpful Tips for Writing

"I can't think of anything."
Start with one part of the problem.
- Can any R's solve it?
- Does it relate to other parts?

"I forgot what we learned."
Look at the front of this page.
- Is any part interesting to you?
- What does the information tell you about it?

"What do I write?"
Think of one thing you can remember from the lessons.
- Is it a part of the problem or a solution?
- What can *you* do to help?

"I don't know what to think about."
Start with something you already do.
- Which R's you do at school?
- Can you also do these at home?

Supplemental Note 2

Scoring Rubric for Posttest

	Points & Descriptions				Score
	3	2	1	0	
Novelty	The response includes ideas that go beyond the information page AND is unique relative to the cohort.	The response includes ideas that go beyond the information page; some ideas are similar to other students within the cohort.	Most of the response simply restates ideas from the information page.	The response is a direct repetition of the information stated on the information page.	
Comprehen-siveness	The response includes at least 3 ideas and elaborates on most of them.	The response includes at least 3 ideas, but does not elaborate on all components (i.e., ideas listed without detail).	The response includes at least 1 idea and elaborates on it.	The response only consists of a single idea as the solution without elaboration.	
Integration of Ideas	Ideas are presented as parts of a "big" solution.	Ideas are presented as a collection of "smaller" solutions.		The response only consists of a single idea as the solution.	
Accuracy of Content Knowledge	The content-based information in the response is accurate.	Most of the information in the response consists of student opinion, but can still be aligned to content.		The information in the response is NOT accurate.	
				Total	
